# The dawn of a new era in cell signalling research

**DOI:** 10.1186/1478-811X-8-7

**Published:** 2010-05-24

**Authors:** Stephan M Feller

**Affiliations:** 1 Cell Signalling Group, Department of Molecular Oncology, Weatherall Institute of Molecular Medicine, John Radcliffe Hospital, Headley Way, Oxford OX3 9DS, UK

## 

Dramatic changes in our thinking of how cells organise and utilise their signal transduction networks are currently arising. These changes have by and large not yet reached the majority of the scientific community and university teaching. Even in the latest editions of top cell biology books the cell signalling machinery is typically depicted as an assembly of fairly unorganised protein molecules, for example diffusing more or less freely in the cytosol. According to current textbook wisdom, upon activation of a signalling pathway its components stochastically meet to generate transient assemblies in the form of signalling 'cascades' or protein complexes with up to 10 or so components. These in turn appear to be linked together into a giant 'floating signalling network' of several thousand proteins which nobody really understands.

We are now beginning to appreciate that this image is far from the truth. It is in fact hindering us in designing more appropriate experiments to understand cell signalling in general and the role of specific components in particular.

Similarly, attempts to describe cellular signalling events with mathematical equations that are based on solution phase diffusion chemistry by self-declared 'systems biologists' [1] are commonly doomed to failure.

A number of recent publications [2-6] and conferences (e.g. the 2009 Seefeld Meeting of the Protein Modules Consortium; http://www.proteinmodules.org/) provide some insight into how we can advance our research field in the future. To give but a few examples:

We must take into serious consideration that signalling mostly occurs in protein assemblies that may be highly organised but are at least specifically localised to distinct, functionally defined subcellular compartments. These complexes are often of considerable size and probably contain vast numbers of components in some cases.

We must take into account that many of the utilised proteins are being produced (translated) in restricted subcellular locations and that they may not diffuse much before they meet most of their interaction partners.

We need to investigate more carefully in which cases signalling enzyme - substrate interactions are primarily driven by highly specific recognition motifs and in which by close proximity of the interacting components [or by a combination of both].

Some signalling proteins appear to be quite scarce, with only a few molecules present per cell, while others can be found in several distinct pools with many thousand copies in each pool. Local signal transduction component ratios within distinct cellular sites therefore deserve much more detailed investigation than is currently undertaken. In this context it should be pointed out that many standard over-expression experiments are rather likely to produce substantial artefacts: the resulting inappropriate amounts and localisations of signalling components will often lead to signal spill-over and/or disrupt the functionality of native complexes.

The distinct signals elicited by different concentrations within the sometimes 3-log-wide physiological concentration range of cytokines and other regulatory factors remains largely unexplored. Too many signalling experiments still rely for convenience on 'super-natural' concentrations of stimuli.

Our knowledge on the temporal features of many signalling events, including oscillations of signals in subcellular compartments of individual cells and waves of signals migrating through tissues is still minimal, especially when physiological concentrations of stimuli are being considered.

We need to explore whether multi-component signalling 'machines' like transmembrane-receptor kinases are indeed pleiomorphic entities [3] which generate 'fuzzy signals' or whether we simply do not understand yet their more sophisticated ways of generating reasonably discrete signalling outputs.

Molecular highways for the directional transport of signalling proteins, as well as meeting points for some molecular signalling components that have not yet been integrated into their destination complexes, are only barely known.

We still have a very limited understanding of how the rather substantial unstructured portion of the proteome, which is often linked to disease development, i.e. not a 'dirty dozen' but the 'dirty thousand', contribute to the organisation and function of cell signalling networks and pathologies [7-10].

I firmly believe that the best is yet to come for the field of biological signal transduction research, but despite impressive technical advances, e.g. HTP-proteomics, -transcriptomics and -genomics, one must still tread carefully when devising conceptual frameworks in which these vast amounts of data are funnelled. If we get these wrong, all those HTP-data will not amount to much in terms of generating robust and realistic information. Moreover, we will require not only fundamental shifts in our currently prevailing concepts but also an arsenal of novel tools in bioinformatics and the 'wet lab'.

Equally important, changing our ways of investigating cellular signalling components, pathways and networks will require abandoning some of the much used current methods, though they may have served us seemingly well in the past. The already mentioned over-expression studies are one example.

On the ultrastructural level, protein crystallography studies will need to be combined more often with analyses by NMR and other solution phase methods to prevent misconceptions from arising. Global folding effects and site-specific long-range effects of introduced point mutations will need to receive more attention. The same is true for the common terminal tagging of proteins, which can lead to mislocalisation or partial unfolding of proteins. In cases where both termini have functional roles, careful tagging in internal loops may be the only option. On the cellular level, monitoring individual cells within a population in real time will become increasingly important.

Eventually, we will even need to think about how different individuals may vary in their signalling components and networks due to their unique genetic compositions. Possibly the most obvious example for this 'personalised' signalling heterogeneity is the tremendous diversity of molecular defects in signalling proteins and networks of human cancers [11-16], but this also applies for some physiological signals transmitted in genetically different individuals.

These are exciting times for young signalling researchers. They will be able to make rapid progress by building on more than three decades of pioneering signal transduction research - if they dare to leave some of the old misconceptions and false dogmas behind. These have often arisen from the 'primitive' tools available at that time and the apparent urge of the human brain to build simple linear models with a small number of components to explain functional relationships.
